# Adaptation to an amoeba host drives selection of virulence-associated traits and genetic variation in saprotrophic *Candida albicans*

**DOI:** 10.3389/fcimb.2024.1367656

**Published:** 2024-03-13

**Authors:** Artid Amsri, Kritsada Pruksaphon, Patcharin Thammasit, Joshua D. Nosanchuk, Sirida Youngchim

**Affiliations:** ^1^ Office of Research Administration, Chiang Mai University, Chiang Mai, Thailand; ^2^ Department of Microbiology, Faculty of Medicine, Chiang Mai University, Chiang Mai, Thailand; ^3^ Department of Medical Technology, School of Allied Health Sciences, Walailak University, Nakhon Si Thammarat, Thailand; ^4^ Center of Excellence Research for Melioidosis and Microorganisms (CERMM), Walailak University, Nakhon Si Thammarat, Thailand; ^5^ Department of Medicine (Division of Infectious Diseases), Department of Microbiology and Immunology, Albert Einstein College of Medicine, New York, NY, United States

**Keywords:** *Candida albicans*, *Acanthamoeba castellanii*, amoeba, microevolution, virulence factor, filamentation

## Abstract

Amoebae are micropredators that play an important role in controlling fungal populations in ecosystems. However, the interaction between fungi and their amoebic predators suggests that the pressure from predatory selection can significantly influence the development of fungal virulence and evolutionary processes. Thus, the purpose of this study was to investigate the adaptation of saprotrophic *Candida albicans* strains during their interactions with *Acanthamoeba castellanii*. We conducted a comprehensive analysis of survival after co-culture by colony counting of the yeast cells and examining yeast cell phenotypic and genetic characteristics. Our results indicated that exposure to amoebae enhanced the survival capacity of environmental *C. albicans* and induced visible morphological alterations in *C. albicans*, particularly by an increase in filamentation. These observed phenotypic changes were closely related to concurrent genetic variations. Notably, mutations in genes encoding transcriptional repressors (*TUP1* and *SSN6*), recognized for their negative regulation of filamentous growth, were exclusively identified in amoeba-passaged isolates, and absent in unexposed isolates. Furthermore, these adaptations increased the exposed isolates’ fitness against various stressors, simultaneously enhancing virulence factors and demonstrating an increased ability to invade A549 lung human epithelial cells. These observations indicate that the sustained survival of *C. albicans* under ongoing amoebic predation involved a key role of mutation events in microevolution to modulate the ability of these isolates to change phenotype and increase their virulence factors, demonstrating an enhanced potential to survive in diverse environmental niches.

## 1 Introduction

Fungal pathogens and their associated infections represent a growing global public health concern, mostly for individuals with health issues or weakened immune systems, including those living with HIV, cancer, or diabetes (Bongomin et al., 2017). Of particular concern is the emergence of antifungal resistance, which threatens our ability to combat these infections. Ecosystem changes might make this problem worse and encourage the evolution of virulence factors in fungi as an adaptation to a changing environment (Wu et al., 2016). The consequences of these developments may have implications for public health, including the potential for an increase in fitness of fungal pathogens and the spread of infectious diseases. A notable example of this is the evolution of environmental strains of *Candida auris* into multidrug-resistant variants, highlighting the severity of this public health issue (Casadevall et al., 2019a; Sharma and Chakrabarti, 2020).

Although *Candida albicans* is commonly considered as a commensal fungus in mammals (Kumamoto et al., 2020), there are increasing numbers of reports on environmental isolates of *C. albicans*. For example, there is clear evidence that certain isolates of *C. albicans* can occupy niches within the natural environment (e.g. fruit, tree bark, soils, and freshwater), which are not linked to mammalian hosts (Opulente et al., 2019; Sautour et al., 2021). Moreover, recent studies have also reported *C. albicans* isolates from agricultural soil forming biofilms, which seem to provide protection against environmental changes (Sidrim et al., 2021). Furthermore, environmental isolates have demonstrated the ability to grow in the presence of high concentrations of various mineral elements (Chaieb et al., 2011). These findings suggest that this fungus possesses unique adaptations that may enhance its pathogenicity.

Based on previous studies, various environmental fungi, including those from the genera *Aspergillus*, *Cryptococcus*, *Histoplasma*, and *Talaromyces*, have undergone evolutionary adaptations that enhance their capacity to cause diseases in humans (De Crecy et al., 2009; Garcia-Solache and Casadevall, 2010; Casadevall, 2012). These adaptations may be a result of interactions with particular hosts, such as rodents, or by their capacity to avoid or resist to the predation by fungivorous soil amoebae in the natural environment (Casadevall et al., 2019b). Previous studies have reported the interaction between *Candida parapsilosis* and *Protostelium aurantium*. Notably, yeast cells exposed to reactive oxygen species (ROS) generated by predators have evolved strategies to respond to this stress (Radosa et al., 2019, 2021). Moreover, several virulence-related traits have been documented in *Saccharomyces cerevisiae* following interactions with amoebae, including an enhanced ability to grow at high temperatures, form pseudohyphae, undergo phenotypic switching, and exhibit resistance to oxidative stress (Koller et al., 2016). Similarly, the interaction between *Cryptococcus neoformans* and *Acanthamoeba castellanii*, a free-living soil amoeba, has revealed significant changes in *C. neoformans* virulence-related traits, with notable implications for its pathogenic potential and capacity to induce severe disease (Steenbergen et al., 2001; Fu et al., 2021). Key virulence factors in *C. neoformans* resistance to predation by *A. castellanii* include capsule size, melanin synthesis, and the release of urease (Chrisman et al., 2011; Fu et al., 2021). These interactions also result in phenotypic changes, chromosomal abnormalities, and DNA sequence mutations in amoeba-passage isolates (Fu et al., 2021). Recently, the interactions of *Candida* spp. or *S. cerevisiae* with *Dictyostelium discoideum* demonstrated that the outcomes of yeast-amoeba interactions could be altered by mutation and other genetic variations (Koller et al., 2016).

In this work, we hypothesized that soil amoebae may play an important role in driving the adaptation and maintenance of virulence factors in environmental strains of *C. albicans*. Thus, our objective was to investigate the evolutionary adaptation of *C. albicans* strains sourced from different environments during their interactions with *A. castellanii* over a period of one-month. Surviving *C. albicans* isolates were observed for phenotypic characteristics, production of virulence factors, and their ability to grow when challenged with various stressors. Moreover, we studied the potential effects of amoeba interactions on the genotypic variations of *C. albicans* by analyzing the whole-genome sequences of selected isolates. Most *Candida* spp. cells have diploid genomes, and genomic evolution results from multiple of genetic events. These events encompass both minor alterations and chromosomal rearrangements that facilitate adaptation to diverse environments, including point mutations, insertions and deletions (indels), and loss of heterozygosity (LOH) events (Mba et al., 2022). For large-scale adaptation, gene dosage variation occurs, which can arise from copy number variants (CNVs). This variability in gene dosage can also manifest at the chromosomal level as aneuploidy. We hypothesized that co-cultivation with *A. castellanii* may contribute to a significant genetic drift, potentially impacting the virulence phenotypes of environmentally isolated *C. albicans*.

## 2 Materials and methods

### 2.1 Organisms and culture conditions

*A. castellanii* strain 30234 was acquired from the American Type Culture Collection (ATCC). This strain was maintained in peptone-yeast extract-glucose (PYG) broth (ATCC medium 354) at 28°C in the dark as detailed in (Steenbergen et al., 2004). For experimental use and routine maintenance, *A. castellanii* was cultured as adherent cells in PYG medium at 28°C for 5-7 days in 75-cm^2^ culture flasks (Bozue and Johnson, 1996). Amoebae were collected by tapping the flasks, centrifuged at 3,000 rpm for 10 minutes, and suspended in sterile phosphate buffer saline (1× PBS, pH 7.2). The cells were counted using a modified Fuchs-Rosenthal chamber, and viability was evaluated by trypan blue staining (Pruksaphon et al., 2022). The initial viability was always greater than 98% (data not shown).

*C. albicans* strain ATCC 90028 and environmental isolates of *C. albicans* were obtained and cultivated according to the directions from Thailand Bioresource Research Center (TBRC), BIOTECH Culture Collection (BCC), Pathum Thani, Thailand. The environmental isolates were collected from different natural ecological niches in Thailand and listed in **Table 1**. The *C. albicans* isolates were stored at -80 °C. Prior to use in experiments, the isolates were grown on Sabouraud dextrose agar (SDA; Difco Laboratories., Detroit, MI) for 24 hours at 28°C. Then, yeast cells were harvested by centrifugation at 5,000 rpm for 15 minutes and washed three times with PBS. The yeasts were then cultivated on peptone yeast extract glucose (PYG) agar at 28°C for 48 hours, collected by centrifugation at 5,000 rpm for 15 minutes, and washed three times with PBS. Cell counting was performed using a hemocytometer prior to their use in all experiments.

**Table 1:** *Candida albicans* isolates.

Genus Species	Strain Isolation	Environmental sources (Abbreviation)
*C. albicans*	TBRC-BCC^a^ 61140	Flower (F)
*C. albicans*	TBRC-BCC 63277	Sediment (S)
*C. albicans*	TBRC-BCC 63603	Mushroom (MH)
*C. albicans*	TBRC-BCC 59444	Water-hot spring (WH)
*C. albicans*	TBRC^b^ 2074	Moss (MS)
*C. albicans*	ATCC^c^ 90028 (Clinical isolate)	–

a Isolate from Thailand Bioresource Research Center (TBRC), Pathum Thani, Thailand.

b Isolate from BIOTECH Culture Collection (BCC), Pathum Thani, Thailand.

c Isolate from American Type Culture Collection (ATCC), USA.

### 2.2 Microscopic assay of *A. castellanii* and *C. albicans* interaction

To study yeast-amoebae interactions, three hundred *C. albicans* yeast cells were spread on PYG agar with a disposable spreader. *A. castellanii* cells (5×10^3^ cells/mL in 5 mL) were poured onto the agar surface containing the yeast cells. The mixture was swirled to ensure complete coverage of the plate by amoebae. Plates were sealed with parafilm and incubated at 28°C for 4 weeks to assess for surviving colonies of *C. albicans* and photographed using a stereomicroscope (Drawell, China). After 4 weeks of interaction, 4 surviving colonies from each strain were randomly picked and transferred into fresh PYG agar without amoeba. The plates were incubated at 28°C for 48 hours, and the macroscopic morphologies of each strain were visualized. The microscopic morphologies were determined under light microscopy (Nikon Eclipse 50i, Tokyo, Japan). Furthermore, colonies of *C. albicans* that interacted with *A. castellanii* for 4 weeks (exposed) were maintained on PYG medium and subsequently investigated for various characteristics by comparison to the control group (unexposed).

### 2.3 Quantification of *C. albicans* viability

To investigate the survival of *C. albicans* (unexposed or exposed isolate) after phagocytosis by amoeba, 1 mL of *A. castellanii*, 10^6^ cells/mL in PBS, was added to a 12-well plate. *C. albicans* yeast cells were then added to the acclimated cultures of *A. castellanii* at a 1:10 effector-to-target ratio and incubated at 28°C for 24 hours. After that, non-phagocytosed *C. albicans* were collected with PBS into a new tube for indirect quantification of yeast cells. The plates were placed on ice for 10 minutes to loosen the amoeba cells from the bottoms of the plates. Then, the inoculated *A. castellanii* was lysed by pulling the suspension through a 27-gauge needle five times or more. The yeast cells were then subjected to a 10-fold dilution. The viability of yeast cells was assessed by spreading them on SDA and incubating at 28°C for 24 hours, determined by the colony-forming units (CFU)/mL count and calculated as direct determination of the cell viability (Pruksaphon et al., 2022).

### 2.4 Tolerance to stressors and antifungal sensitivity test

Yeast cell suspensions were subjected to serial dilutions, and 3 μL droplets of each dilution (10^6^–10 cells/mL) were deposited onto PYG agar supplemented with various stressors at different concentrations. Menadione (MD; Sigma-Aldrich, St. Louis, MO) and hydrogen peroxide (H_2_O_2;_ Merck KGaA, Germany) were utilized to assess resistance to oxidative stressors, while sodium dodecyl sulfate (SDS; Calbiochem, Japan) and Congo red (CR; Sigma-Aldrich, St. Louis, MO) were employed to examine resistance to cell wall stress. The stressors were administered at the following concentrations: 0.025 mM, 0.05 mM, and 0.1 mM for menadione; 1 mM, 2.5 mM, and 5 mM for hydrogen peroxide (Cuéllar-Cruz et al., 2009); 10 μg, 50 μg, and 100 μg for sodium dodecyl sulfate; and 100 μg and 150 μg for Congo red (Schroeder and Ikui, 2019). The azole antifungal drug fluconazole (at 20 and 40 μg/mL) was utilized for antifungal sensitivity testing (Sun et al., 2023). Plates with the various stressors were incubated at 28°C for 24 hours, and the number of colonies were enumerated at a dilution of 10^3^ to determine and compare the colony forming units (CFU)/mL for each strain (Wang et al., 2017). After an incubation period of 48 hours, photographs of plates were taken. To study heat stress, plates were placed at 42 °C for 24 hours, and CFU/mL were then determined.

### 2.5 Germinating assay

To assess yeast germination, 1×10^3^ cells/mL of unexposed *C. albicans* isolates or AC-exposed isolates were inoculated into pooled healthy human serum (Sigma-Aldrich, St. Louis, MO). The inoculated samples were incubated at 37°C for 2 hours, and germlings were counted using a light microscope (Nikon Eclipse 50i, Tokyo, Japan). The percentage of germinated cells was determined by examining 30 fields with each isolate.

### 2.6 A549 cell line and lactate dehydrogenase (LDH) cytotoxicity assay

The A549 cell line (human lung adenocarcinoma cell line, CCL-185™) was obtained from ATCC and cultured in Dulbecco’s minimal essential medium (DMEM; Gibco, Thermo Fisher Scientific, Inc., Waltham, MA) supplemented with 10% fetal bovine serum (FBS; Gibco, Thermo Fisher Scientific, Inc., Waltham, MA) in an incubator at 37°C under 5% CO_2_. The impact of *C. albicans* on A549 cell damage was assessed by measuring LDH release into supernatant. Briefly, yeast cells (control unexposed cells or amoebae-exposed cells) were suspended in Hank’s balanced salt solution (HBSS; Gibco, Thermo Fisher Scientific, Inc., Waltham, MA) supplemented with 1% healthy human serum. Then, confluent monolayers (5×10^5^ cells/mL in a 12-well plate) were inoculated with each isolate of *C. albicans* at an optimized MOI of 5. After 12 hours of infection, the culture supernatants were centrifuged to remove yeast cells and debris, and LDH enzymatic activity was measured using a Cobas 8000 automatic analyze (Roche Diagnostics, Mannheim, Germany) (Owen et al., 2015). The LDH positive control was established by lysing the uninoculated A549 cells with 0.5% Triton X-100 in HBSS. The enzymatic activity of supernatant from uninoculated control A549 cells was normalized as the background level. The percentage of A549 cytotoxicity was calculated following formula: 


LDH cytotoxicity(%)=(LDH activity in infected cells/LDH activity in control cells)×100

### 2.7 Whole genome sequence analysis

Single colonies from unexposed (UN) and exposed (EX) isolates of *C. albicans* TBRC-BCC 59444 were isolated on PYG medium. Three colonies from each isolate (UN and EX) were individually inoculated into 5 mL of YPG medium and allowed to grow overnight at 30°C. The resulting cultures were designated as EX1, EX2, EX3, UN1, UN2, and UN3 isolates. The DNA of each isolate was extracted as detailed in (Wangsanut et al., 2023).

DNA samples, each containing 6 μg or more, were shipped to the Novogene service for whole-genome DNA sequencing using the Illumina Sequencing Package. The DNA sequencing data were returned in FASTQ (.fq) format file, containing sequencing reads and corresponding base quality. The data was then converted to sorted BAM format using Samtools (Li et al., 2009). To analyze results, paired-end reads were mapped to the reference genome of *C. albicans* SC5314 using Yeast Mapping Analysis Pipeline (YMAP) (Abbey et al., 2014). Additionally, Single nucleotide polymorphisms (SNPs) and insertion-deletion (indel) variations were investigated by GATK (DePristo et al., 2011). Copy-number variation (CNV) and Structural variants (SVs) were detected by CNVnator (Abyzov et al., 2011) and BreakDancer (Chen et al., 2009), respectively. Identified mutations were individually assessed using the Integrative Genomics Viewer (IGV) (Ene et al., 2018).

### 2.8 Biofilm formation assay

To determine the formation of biofilm in amoeba-exposed compared to unexposed yeast, a biofilm formation assay was performed using a *C. albicans* isolate from a water-hot spring (WH) according to the protocol described (Gulati et al., 2018). Briefly, *C. albicans* isolates grown for 48 hours on PYG were diluted in RPMI-1640 to obtain 5×10^4^ cells/mL. Subsequently, 100 μL of *C. albicans* (5×10^3^ cells/well) were seeded into a 96-well plate and incubated at 37 °C for 24 hours. Afterward, the plate was washed three times with PBS and fixed with 99% (*v/v*) methanol. The plate was then air-dried at room temperature for 45 minutes. After that, the biofilm was stained with 0.05% crystal violet (CV) for 30 minutes and washed three times with PBS. To measure absorbance, 200 μL of 33% (*v/v*) acetic acid in PBS was added to de-stain for 45 minutes, and one-hundred microliter of each well was transferred to a new plate and measured at OD_595_ nm.

### 2.9 Determination of growth in various iron conditions

To investigate the impact of the *FTH2* gene mutation on the growth of exposed isolates compared to unexposed isolates under iron-free, iron-depleted, and iron-replete conditions (Hsu et al., 2011). To generate each iron condition, PYG medium was supplemented with 100 μM bathophenanthroline (BPS) to chelate iron ions (Fe^2+^ or Fe^3+^) in the media which is an iron-free condition. For the iron-depleted condition, PYG medium was supplemented with 100 μM BPS and 0.01 mM ferric ammonium citrate (FAC). In the iron-repletion, 0.1 mM FAC was added to the medium. In brief, yeast cells were cultured on PYG medium at 28 °C for 48 hours. After that, the cells were counted to obtain 10^6^ cells/mL and subjected to a 10-fold dilution, ranging from 10^6^ to 10^1^. *Candida* cells were spotted on the medium conditions and incubated at 28 °C for 24 hours. The number of colonies were counted at a dilution of 10^3^ to determine and compare the colony forming units (CFU)/mL for each isolate.

### 2.10 Investigation of the growth of *C. albicans* on the medium with or without adenine

To evaluate the growth of an adenine auxotrophic *C. albicans* strain with an *ADE4* gene mutation, a synthetic complete (SC) medium with or without adenine was used. SC medium was prepared by supplementing with 2% dextrose, 6.7% yeast nitrogen base (YNB) plus ammonium sulfate, and with 0.2 mM adenine (SC+Ade) or without adenine (SC-Ade) (Wangsanut et al., 2017). Briefly, the yeast cells were cultured on PYG medium at 28 °C for 48 hours. Subsequently, the cells were counted using a hemocytometer. The suspended cells were then subjected to a 10-fold dilution ranging from 10^6^ to 10^1^. To observe the growth of *Candida* colonies, these cells were spotted onto SC+Ade or SC-Ade medium and incubated at 28 °C for 24 hours.

### 2.11 Statistical analysis

The data underwent statistical analysis utilizing a range of tests. The normality of the dataset was assessed using Shapiro-Wilk test. Virulence-related phenotypic characteristics between unexposed and exposed-*Candida* were compared using Kruskal-Wallis non-parametric test. Growth at 42 °C was determined by one-way ANOVA. Two-way ANOVA with Tukey’s multiple comparison test was used to compare other experiments. A significance threshold of *P* < 0.05, *P* < 0.001, and *P* < 0.0001 was adopted for all statistical analyses. The data analysis was conducted using GraphPad Prism 9.0 software (version 9.0) and IBM SPSS Statistics (version 29).

## 3 Results

### 3.1 Selection of amoeba resistance strains and investigating the impact of amoeba co-cultivation on the phenotype

**Figure 1:**
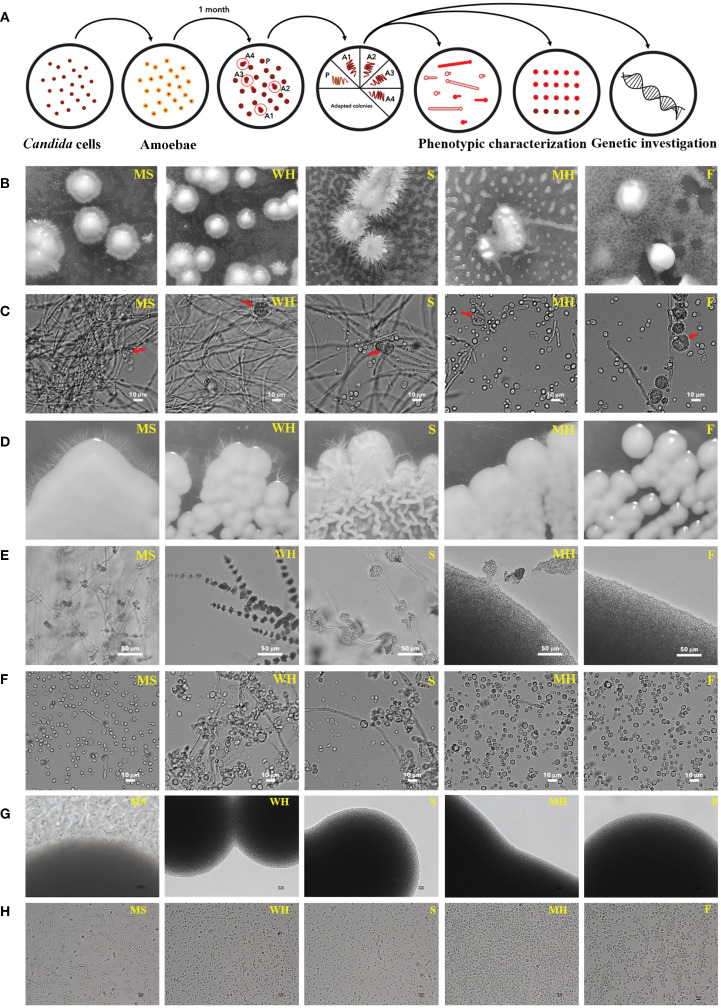
Colony morphologies of *C. albicans* following co-incubation with amoebae in PYG medium; schematic of experimental setup for amoeba-exposed isolate selection. **(A)** Approximately 300 yeast cells of each *C. albicans* isolates were initially plated on agar. Approximately 25,000 *A. castellanii* cells were introduced to the agar with *C. albicans*. After 4 weeks of co-incubation, colonies emerged within the predation zone of *A. castellanii*. Four colonies were randomly chosen for each isolate and individually transferred to fresh medium. Following this, the four colonies of each isolate were subjected to phenotypic characterization. Control colonies were obtained from plates for each isolate where the yeasts were grown without amoebae. **(B)** Location of surviving colonies (after 4 weeks): Stereoscopy of surviving colonies of each *C. albicans* strains were exhibited within a mat of amoebae. **(C)** Microscopic visualization of surviving colonies: The cells within the surviving colonies displayed hyphae, pseudohyphae, and yeast morphologies, 400× magnification (the red arrow represents an amoeba cell). **(D, E)** Subsequent culture and monitoring: Surviving *C. albicans* were isolated and individually transferred to fresh PYG medium, and sub-cultured through four passages. The isolates were monitored for their colony characteristics over a three-day period at 28°C. MS, WH, and MH isolates exhibited smooth colonies and produced hyphae around their colonies, whereas S isolates displayed serrated colonies and hyphae. The F isolate formed smooth colonies without hyphae. The colonies were visualized at 200× magnification. **(F)** Yeast cells in wet mount: Yeast cells cultivated without amoebae were identified in wet mount of samples taken from the colonies of each isolate. Smooth colonies were formed primarily from these yeasts and pseudohyphal cells, while serrated colonies consisted of yeast, pseudohyphal cells, and long filaments. The wet mounts were viewed at 400× magnification. **(G)** Unexposed isolates were grown on a fresh PYG medium and monitored for colony characteristics over a three-day period at 28°C. WH, S, MH, and F isolates produced yeast colonies without hyphae around their colonies, whereas MS colonies produced hyphae (magnification 200×). These figures were used as a control for comparison to **Figure 1**, **(E, H)** Yeast cells in wet mounts: Unexposed isolates were identified in wet mounts of samples taken from the colonies of each isolate. All isolates presented yeast cells, but the MS isolate produced yeast and filamentous forms (magnification 400×). These figures were used as a control for comparison to **Figure 1**, **(F)** The sources of the abbreviations MS, WH, S, MH and F are described in **Table 1**.

We investigated the interaction between five environmental isolates of *C. albicans* (from flower; F, sediment; S, mushroom; MH, water-hot spring; WH, moss; MS) and *A. castellanii*. The experimental approach involved co-culturing these organisms on PYG agar and is detailed in **Figure 1A**. The co-cultures were maintained for 4 weeks, at which time small colonies of yeast were macroscopically visible, predominantly situated beneath the amoebae mat (**Figure 1B**). Microscopic examination of *C. albicans* surviving colonies revealed a mixed population comprised by yeast, pseudohyphal, and hyphal forms in all isolates of environmentally derived *C. albicans*, with varying proportions among each individual isolate (**Figure 1C**).

To ensure a representative sampling, four single colonies were randomly selected from each isolate to study their phenotypic characteristics and transferred onto fresh PYG agar plates devoid of amoebae. Unexposed isolates were used as controls. Within a span of 24 hours, discernible colonies composed exclusively of yeast cells appeared on the agar surface. Importantly, after a period of 3 days of agar growth, colonies from *C. albicans* MS, WH, S, and F were smooth while colonies of S were rough (**Figure 1D**). Moreover, we found that four isolates (MS1-4, WH1-4, S1-4 and MH1-4) generated hyphae following their interaction with amoebae (**Figures 1D–F**). Notably, unexposed *Candida* cells from each isolate exhibited a normal yeast morphology (**Figures 1G, H**). Even though isolates MH1-4 presented yeast colonies, they also generated segments with hyphal morphology around their colonies (**Figures 1D–F**). However, there was a single MS isolate that spontaneously germinated to form hyphae, even though it had not been exposed to amoebae (**Figures 1G, H**). The experiment was subsequently replicated utilizing *C. albicans* ATCC 90028. However, no surviving colonies were observed from the control strain on three co-incubated plates with amoebae even after an additional week (4-weeks) of incubation. These findings provide compelling evidence that, following interaction with amoebae, the environmental strains were capable of displaying a diverse array of cellular and colony morphologies, even in the absence of amoebae in the growth medium.

### 3.2 Effects of amoeba exposure impact on *C. albicans* virulence factors and survival

**Figure 2:**
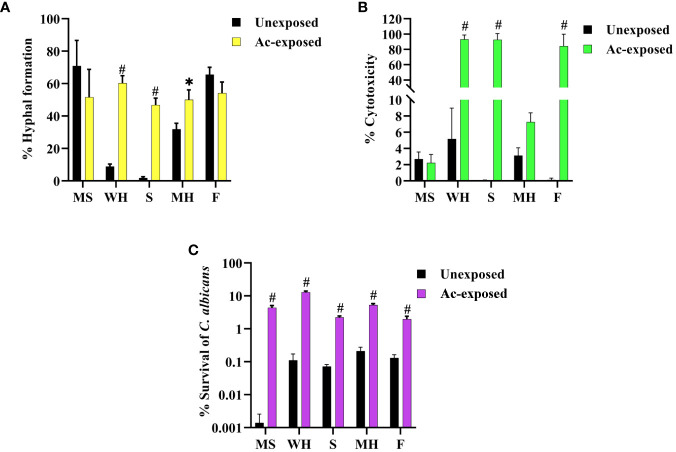
Impact of amoebae interaction on *C. albicans* virulence factors and survival. The effects of co-culture with amoebae on the *C. albicans* germination in serum, cytotoxicity to A549 cells, and survival rate were studied. **(A)** The germination experiment was conducted with independent triplicates, with a minimum of 1,000 cells counted for exposed isolates; the unexposed isolate served as a control. **(B)** The effects of amoebae on the capacity of *C. albicans* to damage A549 cells was also assessed. LDH release was used to compare the cytotoxicity of A549 cells inoculated with both exposed and unexposed isolates. The LDH control positive was established by lysing A549 cells cultivated without yeast cells using 0.5% Triton X-100, and the negative control was A549 cells grown in standard medium. **(C)** The percentage of *C. albicans* survival after phagocytosis by amoeba was determined, comparing between amoebae-exposed and unexposed isolates. Data represent the means ± standard deviation (SD) from three independent experiments. Asterisk (*) and hash (#) signify data that significantly differed at *P<* 0.05 and *P<* 0.0001 based on a Kruskal-Wallis non-parametric test. Amoebae-exposed represents yeast cells co-incubated with amoebae whereas unexposed represents yeast cells growth without amoebae.

Our prior findings revealed that interaction with amoeba results in the alteration of *C. albicans* morphologies. We speculated that this change might lead to an increase in the pathogenicity of the fungus. Thus, we next investigated the impact of amoebae interaction on the pathogenicity of *C. albicans*, particularly focusing on hyphal formation and invasion of the lung human A549 cell line. To achieve this, we analyzed various virulence-related phenotypic characteristics of isolates derived from five *C. albicans* isolates. We observed that exposed isolates WH, S, and MH exhibited significantly faster and greater amounts of hyphal formation compared to their respective unexposed strains when cultured in healthy human serum within 2 hours after incubation. In contrast, exposed isolates MS and F did not show differences in hyphal formation when compared to their unexposed isolates (**Figure 2A**). Next, to assess fungal-associated cytotoxicity, we measured the release of LDH by the A549 cells (Saiprom et al., 2023). We investigated whether this cytotoxicity was present during interactions with host-exposed *C. albicans* isolates. After inoculating the exposed *C*. *albicans* isolates and their unexposed isolates with A549 cells, we measured LDH enzyme release at 12 hours post-incubation. The result showed that the unexposed strain resulted in 5% cytotoxicity. Exposed MS and MH exhibited cytotoxicity percentages similar to their unexposed, ranging from 2% to 5%. On the other hand, a high level of LDH was detected from A549 cells inoculated with exposed WH, S, and F, with levels up to 99% (**Figure 2B**). Moreover, we assessed the percentage survival of exposed isolates compared to that of their unexposed strains after being phagocytosed by amoebae. The results showed that the unexposed strains had a low survival rate, whereas the exposed isolates exhibited a higher percentage of survival (**Figure 2C**). Our findings suggest that the WH and S evolved a heightened ability to generate hyphae and invade and destroy host cells. Although the exposed F had a percentage of hyphal formation similar to its unexposed counterpart, it displayed a remarkable capacity to cause damage to A549 cells. This interesting characteristic suggests that this particular isolate might be able to produce elevated levels of *Candida* exotoxins or enzymes compared to its unexposed. However, further studies are necessary to elucidate the specific factors responsible for the virulence of the F isolate.

### 3.3 Amoeba - C*. albicans* co-cultivation affects the resistance of yeast cells against various stressors

To investigate whether cultivation with amoebae affected the growth of the *C. albicans* isolates via stressor responses, the fungal isolates were subjected to various individual stress conditions. These conditions included exposure to differing concentrations of oxidative stresses (H_2_O_2_ and menadione), cell wall stresses (Congo Red and SDS), and thermal stress at 42°C. Growth was assessed on plates at a concentration of 10^3^ cells, and the average growth from six experiments (two independent technical replicates for each of three biological repeats) was used as a quantitative measure to compare the effects of stress on growth of each isolate compared to its growth in standard media.

**Figure 3:**
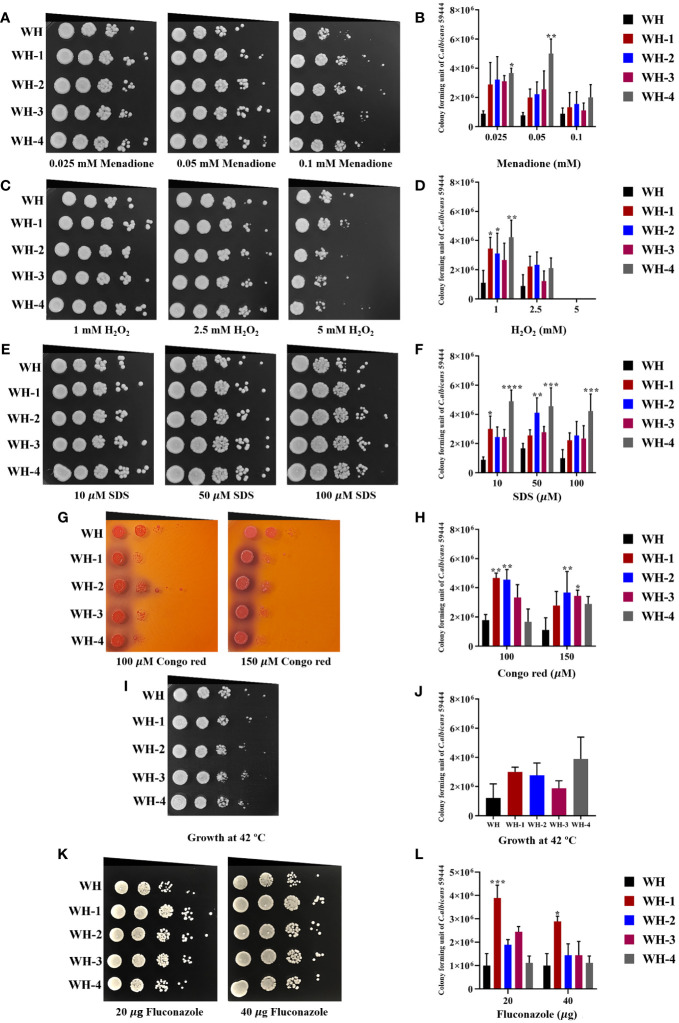
Comparison of *C. albicans* isolates cultivated with or without amoebae to subsequent challenges with various stressors. The growth of amoebae-exposed and unexposed isolates under menadione **(A, B)** and hydrogen peroxide (H_2_O_2_) **(C, D)** as oxidative stress, sodium dodecyl sulfate (SDS) **(E, F)** and Congo red **(G, H)** as cell wall stress, incubation at 42°C as thermal stress **(I, J)**, and fluconazole **(K, L)** as antifungal agent. Cells were 10-fold serially diluted (10^6^ to 10^1^ cells/mL) and spotted onto a PYG medium containing various concentrations of menadione or hydrogen peroxide and grown for 24 hours at 28°C. Colony forming units (CFU)/mL were then determined at a dilution of 10^3^ cells/mL. Colonies were photographed after 48 hours of inoculation Amoeba-passaged isolates are labeled with numbers preceded by the letters WH1-4 to indicate their origin from isolates WH. Asterisks *, **, ***, and hash (#) indicate significant differences at *P*< 0.05, *P*< 0.01, *P*< 0.001, and *P<* 0.0001, based on ordinary one-way or two-way ANOVA in conjunction with Tukey’s multiple comparison test.

Isolates were tested in various concentrations of H_2_O_2_ and menadione, the latter being a cytotoxic quinone that generates superoxide (Frohner et al., 2009). Results showed that the growth of WH1-4 and other isolates was similar to control conditions at concentrations of 1 and 2 mM of H_2_O_2_, but not at 5 mM (**Figures 3C, D**, **Supplementary Figure 1**). Moreover, the amoebae-exposed isolates survived menadione up to a concentration of 10 μM (**Figures 3A, B**, **Supplementary Figure 1**), marking them as the most resistant to superoxide ion. When the number of colonies for each sample was calculated, the results show that amoebae-exposed isolates exhibited significantly enhanced growth under both oxidative stressors compared to their unexposed isolates. Moreover, all amoebae-exposed isolates showed increased growth when exposed to thermal stress at 42°C, especially S4, WH4, MH1-4, F1, F3, and F4 (**Figures 3I, J**, **Supplementary Figure 1**). Most of the amoebae-exposed *C. albicans* strains demonstrated an increased resistance to SDS (**Figures 3E, F**). In contrast, amoebae-exposed several isolates were sensitive to Congo Red and could not grow under this stressor but isolates WH1-4 and MH2 grew in the presence of Congo Red (**Figures 3G, H**, **Supplement Figure 2**). Furthermore, these amoebae-exposed isolates exhibited a halo zone around their colonies on Congo Red, a trait absent in the unexposed isolate. These results demonstrated the substantial influence of interactions between amoebae and *C. albicans*. Such interactions significantly impact the adaptive resistance of *C. albicans* to oxidative agents, cell wall stressors, and high-temperature challenges.

### 3.4 Amoebae-exposure can alter *C. albicans* fluconazole susceptibility.

We further investigated the impact of amoebae on the growth of the amoebae-exposed yeast in the presence of an antifungal agent. We assessed changes in drug susceptibility by performing spot assays, comparing the amoebae-exposed isolates to their unexposed counterparts for growth in the presence of fluconazole. All unexposed isolates were susceptible to 20 μg/mL and 40 μg/mL of the antifungal drug. Interestingly, although many of the exposed isolates remained susceptible to fluconazole, isolates WH1, S1, S2, and S4 exhibited a prominent increase in resistance to fluconazole compared to their unexposed counterpart (**Figures 3K, L**, **Supplementary Figure 1**). These findings demonstrate that interactions with amoebae may induce adaptations in *C. albicans* that alter their susceptibility to fluconazole.

### 3.5 Genotypic variations changes in *C. albicans* occur in response to amoeba interaction

In a previous study on *C. neoformans* host adaptation, genotypic variations were shown to play a role in altering morphology and virulence (Magditch et al., 2012). Thus, we investigated whether amoeba interactions could have an impact on genotypic variation in an environmental *C. albicans* strain. To achieve this, we conducted a comprehensive analysis of the *C. albicans* genome for an amoebae-exposed WH isolate and its unexposed counterpart. The total length of the SC5314 assembled genome sequence was 14,282,666 base pairs, while the mapping rate of unexposed and exposed isolates range from 92.15% to 94.61%. The average depths were between 52.55 and 88.51, and coverages range were from 99.68% to 99.74%. These results were in the qualified normal range. Thus, we examined single nucleotide polymorphisms (SNPs), insertion-deletion (Indels), copy number variation (CNVs), and structural variation (SVs), and utilized the reference genome of *C. albicans* SC5314 for comparisons. The resulting genome-wide scale changes were analyzed by YMAP plots (**Supplementary Figure 2**).

#### 3.5.1 Single nucleotide polymorphisms

**Figure 4:**
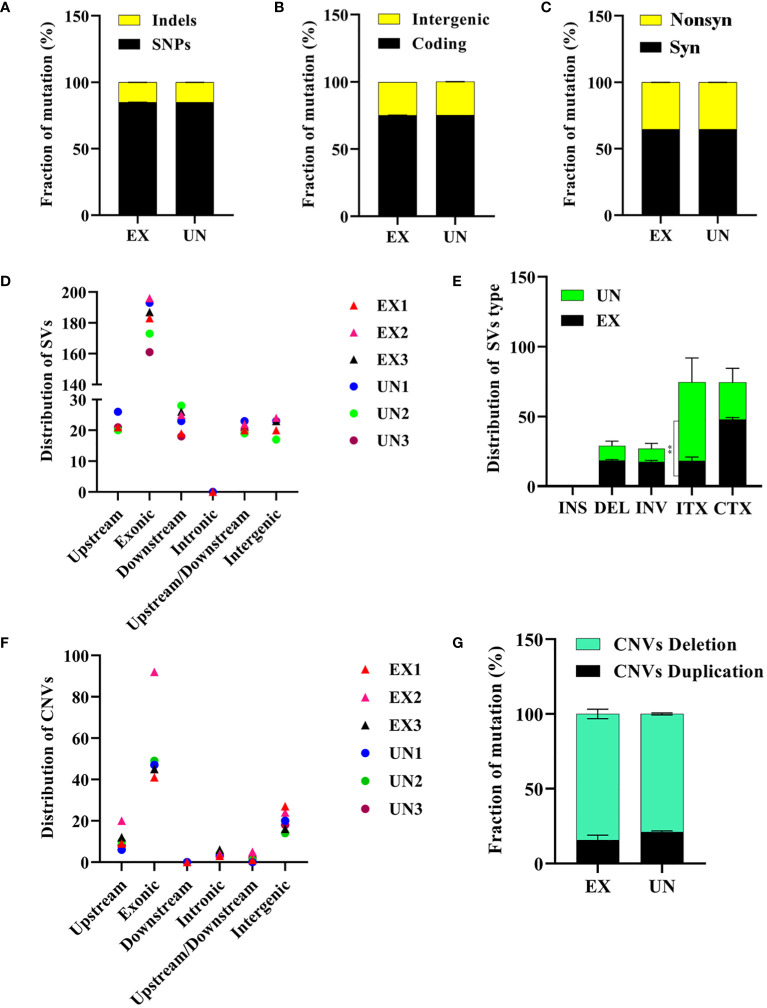
Analysis of SNPs, Indels, structural variations and copy number variations in *C. albicans* genomes after amoeba interaction. **(A)** Distribution of SNPs and Indels, **(B)** intergenic and coding mutations, **(C)** synonymous and nonsynonymous mutations. **(D)** Distribution of structural variation (SV) in different regions and **(E)** distribution of structural variation (SV) types were shown. The intra-chromosomal translocations (ITX) distribution was significantly different between exposed and unexposed isolates (*P=*0.0031). **(F)** Distribution of copy number variation (CNV) in different regions and **(G)** distribution of copy number variation (CNV) types are also presented. ** in E signifies significance. Noted that panels indicate mutations resulting from amoebae-exposed isolate (EX), compared with the unexposed (UN) isolate.

Genome sequencing analysis revealed that the unexposed isolate exhibited a ratio 156,904 (85.03%) of SNPs and 27,397 (14.97%) of Indels. Similarly, the amoebae-exposed isolate displayed 156,815 (85.07%) of SNPs and 27,486 (14.92%) of Indels. The number of SNPs and Indels in the amoebae-exposed strain was 75.17% in a coding region and 24.64% in an intergenic region, which closely matched those of its unexposed (**Figures 4A, B**, **Supplementary Tables 1, 2**). Among the identified SNPs, two events were annotated as high-impact mutations, resulting in disruptions of the coding region, stop-gain, and frameshift mutations. However, the distribution of SNPs in the amoebae-exposed isolate showed that 64.68% were synonymous SNPs, while 35.31% were non-synonymous SNPs. The numbers of synonymous and non-synonymous SNPs in the unexposed strain were not different from those in the exposed isolate (**Figure 4C**). Despite the similar number of SNPs between the two groups, the positions where the SNPs occurred in the amoebae-exposed isolate were different compared to the unexposed.

Notably, a nonsynonymous SNP change was identified (**Table 2**, **Supplementary Table 6**) in the CAALFM_C107770WA gene (*FGR6-3*), which encodes a filamentous growth regulator protein. This SNP involved substitutions from nucleotide base C to A and G to A at positions 1,692,063 and 1,692,150, respectively, resulting in a missense mutation that replaced leucine 258 (Leu258) with isoleucine (Ile) in EX1 and EX2, and valine 287 (Val287) with isoleucine in EX3. Another SNP (C to T; glutamine, Gln256*) was identified in *FGR6*-*3*, leading to an early stop codon (UAG) in the EX2 isolate. Furthermore, we found missense SNPs in CAALFM_C210870WA, CAALFM_C301130CA, and CAALFM_CR09700WA across all amoebae-exposed isolates. Although these genes have not been extensively characterized, these SNPs resulted in the change of arginine 320 (Arg320) to lysine (Lys), Ile142 to asparagine (Asn), and threonine 367 (Thr367) to isoleucine, respectively. Also, missense SNPs were observed in the *CIP1* gene, encoding an oxidoreductase, in both EX2 and EX3 isolates. These SNPs led to changes, including the alteration of Gln128 to Lys. Similarly, the *ALS2* and *ALS4* genes, encoding adhesin proteins, exhibited missense SNPs in the same isolates, resulting in the alteration of alanine 2081 (Ala2081) to aspartic acid (Asp) and histidine 2085 (His2085) to tyrosine (Tyr), respectively.

**Table 2:** High- and moderate-impact SNPs and indels found exposed isolates.

Gene identifier	Gene function	Isolates	Chromosome	Position	Reference	Alternate	Impact
**CAALFM_C100060WA**	**TUP1; Transcriptional corepressor**	**EX1, EX2, EX3**	**NC_032089.1**	**12493**	**GCAA**	**G**	**Inframe deletion**
**XM_711448.2**	**ADE4; Phosphoribosylpyrophosphate amidotransferase**	**EX1, EX2, EX3**	**NC_032089.1**	**1677739**	**CTCATCAT**	**CTCAT**	**Conservative inframe deletion**
**CAALFM_C107770WA**	**FGR6-3 pseudo; Filamentous Growth Regulator**	**EX2**	**NC_032089.1**	**1692057**	**C**	**T**	**Stop gain**
**CAALFM_C107770WA**	**FGR6-3 pseudo; Filamentous Growth Regulator**	**EX1, EX2**	**NC_032089.1**	**1692063**	**C**	**A**	**Missense**
**CAALFM_C107770WA**	**FGR6-3 pseudo; Filamentous Growth Regulator**	**EX3**	**NC_032089.1**	**1692150**	**G**	**A**	**Missense**
**XM_717036.1**	**Hypothetical protein**	**EX3**	**NC_032089.1**	**2805118**	**TCCCCCCCCCCCC**	**TCCCCCCC**	**Frameshift**
**XM_709981.1**	**Hypothetical protein**	**EX1, EX2, EX3**	**NC_032089.1**	**3185577**	**GT**	**G**	**Frameshift**
**XM_712079.2**	**Fungal-type vacuole membrane localization**	**EX1, EX2, EX3**	**NC_032090.1**	**2228219**	**G**	**A**	**Missense**
**XM_713440.2**	**ZZ-type zinc finger protein**	**EX1, EX2, EX3**	**NC_032091.1**	**229638**	**A**	**T**	**Missense**
**XM_715404.2**	**RGT1; Zn(II)2Cys6 transcription factor; transcriptional repressor**	**EX1, EX2**	**NC_032092.1**	**526632**	**CAACAACAAAAACAACAA**	**CAACAACAA**	**Disruptive inframe deletion**
**XM_705568.2**	**CDS1; phosphatidate cytidylyltransferase**	**EX3**	**NC_032093.1**	**914097**	**C**	**T**	**Stop gain**
**XM_709227.2**	**CIP1; Oxidoreductase**	**EX2, EX3**	**NC_032094.1**	**209928**	**G**	**T**	**Missense**
**XM_709227.2**	**CIP1; Oxidoreductase**	**EX2, EX3**	**NC_032094.1**	**209940**	**G**	**T**	**Missense**
**XM_705333.2**	**ALS4; GPI-anchored adhesin protein**	**EX2**	**NC_032094.1**	**898502**	**G**	**A**	**Missense**
**XM_707553.2**	**ALS2; ALS family protein**	**EX3**	**NC_032094.1**	**977456**	**C**	**A**	**Missense**
**XM_713605.1**	**TLO1; Telomere-proximal protein**	**EX1, EX2**	**NC_032096.1**	**9463**	**A**	**C**	**Missense**
**XM_712825.2**	**FTH2; Iron transporter protein**	**EX1, EX2, EX3**	**NC_032096.1**	**304291**	**TTAGAACTAGAACTAGAACTA**	**TTAGAACTAGAACTA**	**Conservative inframe deletion**

Furthermore, the *TLO1* gene, encoding a telomere-proximal protein, showed a missense SNP, leading to the substitution of glutamic acid 118 (Glu118) with Ala. We identified a SNP in the CAALFM_C504100WA gene (*CDS1*) of the EX3 isolate. This SNP resulted in a stop-gain mutation at position 914097 (C to T; glutamine, Gln98*), causing a premature termination codon (UAA). *CDS1* encodes a phosphatidate cytidylyltransferase, which plays a crucial role in membrane lipid and cell wall component synthesis in both *C. albicans* and *S. cerevisiae* (Uhl et al., 2003). Additionally, we found a frameshift mutation in the XM_717036.1 gene of EX3 isolate, which encodes to the non-LTR retrotransposon protein family (Goodwin et al., 2001) as well as the XM_709981.1 gene, encoding a DNA binding domain protein, which was found in amoebae-exposed isolates. These results demonstrated that the amoebae-exposed strains presented high and moderate-impact mutations, which were found in specific genes after amoebae interaction. Missense SNPs were observed in genes that related to filamentous growth regulation, oxidoreductase activity, adhesin proteins, and telomere-proximal proteins. Particularly, a stop-gain mutation was identified in a gene, encoding a phosphatidate cytidylyltransferase, which is important for membrane lipid and cell wall component synthesis. Frameshift mutations were found in genes related to retrotransposon proteins and DNA binding domain proteins.

#### 3.5.2 Nucleotides insertion-deletions

In addition to SNPs affecting gene mutations, we observed the presence of insertions and deletions (Indels) influencing mutations in the genomes of amoebae-exposed isolates (**Table 2**, **Supplementary Table 6**). Notably, these Indels were identified in the CAALFM_C100060WA (*TUP1*) gene. *TUP1* encodes a transcriptional repressor critical for negatively regulating filamentous growth in *C. albicans* (Kebaara et al., 2008). In the amoebae-exposed isolate, the Indel was characterized by the deletion of CAA within the exonic region of the *TUP1* gene. This deletion resulted in an in-frame deletion of glycine in all exposed isolates. Furthermore, we observed in-frame deletions in other genes, including XM_711448.2 (*ADE4*), which encodes phosphoribosylpyrophosphate amidotransferase; XM_715404.2 (*RGT1*), which encodes a Zn(II)2Cys6 transcription factor acting as a transcriptional repressor in controlling sugar transport and metabolism in *C. albicans* (Sexton et al., 2007); and XM_712825.2 (*FTH2*), which encodes an iron permease involved in iron acquisition (Mochochoko et al., 2021). These in-frame deletions let to the loss of Ile in *ADE4* for amoebae-exposed isolates, Lys and Thr in *RGT1* for EX1 and EX2 isolates, and Thr and Arginine (Arg) in *FTH2* for all exposed isolates. These findings have revealed the presence of insertions and deletions (Indels) in genes such as *TUP1*, *ADE4*, *RGT1*, and *FTH2*. These mutations have been observed to have a substantial impact on the function of transcriptional repressors and might affect to their proteins responsible for AMP biosynthesis, sugar transport, and iron metabolism.

Particularly, interaction with amoebae resulted in mutations in the *FTH2* and *ADE4* genes, potentially affecting iron metabolism and AMP biosynthesis, respectively. The impact of the *FTH2* gene mutation on *Candida* growth in various iron conditions was assessed. Our results revealed that amoebae-exposed isolates with a mutation in the *FTH2* gene could grow under iron-free, iron-depletion, and iron-repletion conditions, showing no significant difference compared to unexposed isolates (**Supplementary Figure 3**). Furthermore, we found that exposed isolates, defective in the *ADE4* gene, could grow on medium without adenine (**Supplementary Figure 4**). These findings suggest that mutations in the *FTH2* and *ADE4* genes might not have an impact on iron metabolism and AMP synthesis in *C. albicans*.

#### 3.5.3 Chromosomal abnormalities

Finally, we investigated the potential impact of amoeba interactions on chromosomal abnormalities in exposed isolates. We conducted an analysis of structural genome variations and chromosomal copy number variations (Selmecki et al., 2010) in the amoebae-exposed isolates compared to the unexposed. Our analysis focused on the distribution of SVs, which were predominantly found in exonic regions across all isolates (**Figure 4D**). We observed intra-chromosomal translocations (ITX) in the amoebae-exposed isolates, showing 17.67% of the total SVs (138 out of 687). This percentage was lower than that observed in the unexposed isolate. In contrast, inter-chromosomal translocations (CTX) were present in approximately 46.28% of the total SVs (356 out of 687) in the amoebae-exposed isolate, similar to the unexposed isolate. Additionally, inversions (INV) and deletions (DEL) were detected in approximately 17.56% (135 out of 687) and 18.49% (142 out of 687) of the amoebae-exposed isolate’s SVs, respectively, both of which were higher than those in the unexposed isolate (**Figure 4E**, **Supplementary Tables 3, 4, 7**).

Our analysis of CNVs revealed variations among the isolates, with CNVs predominantly located in exonic regions (**Figure 4F**). Unexposed isolates displayed 21.12% duplications (16 out of 77) and 78.87% deletions (61 out of 77). In contrast, the amoebae-exposed isolates exhibited a higher percentage of CNV deletions, showing 84.26% (87 out of 102), particularly with EX2 displaying a significant increase in chromosomal deletions compared to the other isolates (**Figure 4G**, **Supplementary Table 5**).

**Figure 5:**
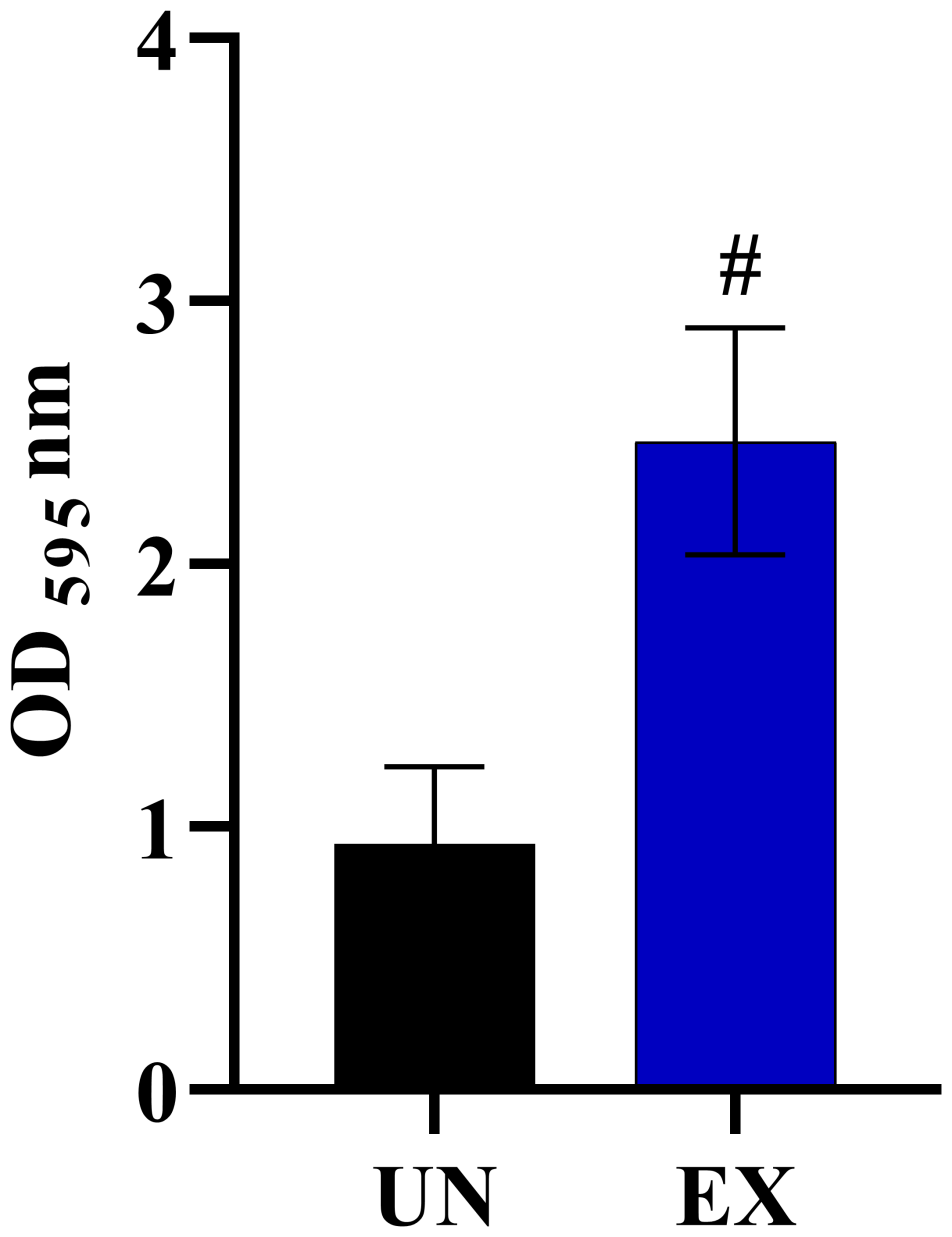
The impact of amoeba interaction on *Candida albicans* (WH) biofilms using crystal violet staining. Biofilm was formed at 24 hours of incubation and absorbance was measured at OD_595_ nm, comparing *C. albicans* previously cultivated in medium alone (UN) with amoebae-exposed (EX) isolates. Data are represented as mean ± SD from three independent experiments. # indicates *P<*0.0001.

We also identified vital chromosomal duplications in the amoebae-exposed isolates, specifically involving chromosome NC_032090.1 and NC_032091.1, while no such duplications were detected in the unexposed isolates (**Table 3**). These duplications covered genes of particular interest, including *FGR6*-*4*, *MET1*, CAALFM_C202950WA on chromosome NC_032090.1, as well as CAALFM_C300030CA on chromosome NC_032091.1. These genes encode protein that play essential roles in filamentation (Uhl et al., 2003), methionine biosynthesis, NAD biosynthesis, and colony morphology regulation, respectively. The analysis results also revealed deletion regions in several chromosomes, i.e. NC_032089.1, NC_032091.1, NC_032092.1, NC_032093.1, NC_032094.1, and NC_032096.1. A detailed summary of these deletions is provided in **Table 3** and the **Supplementary Table 8**. Notably, chromosome NC_032089.1 exhibited deletions in 14 regions, potentially impacting gene stability, with the most remarkable effects observed in isolate EX2. Interestingly, the specific position in chromosome NC_032089 affected about five genes, as shown in **Table 3**. These genes are involved in biofilm formation, which suggests a potential role of this deletion in altering the biofilm-related characteristics of the strain. Additionally, we observed CNVs of the deletion type in chromosome NC_032091.1, affecting genes such as CAALFM_C301250WA and CAALFM_C307020WA. These genes encode proteins related to biofilm formation and hyphal growth regulation, respectively. A Crystal Violet assay was performed to investigate whether the deletion type of CNVs in genes related to biofilm synthesis may influence the level of biofilm. The results revealed significantly higher biofilm levels in amoebae-exposed isolates compared to unexposed isolates (*P*<0.0001) (**Figure 5**). Hence, this finding indicated that these related genes might not be directly involved in controlling biofilm regulatory pathways.

**Table 3:** Impact SVs and CNVs found in exposed isolates.

Gene identifier	Gene function	Isolates	Chromosome	Position	Impact	Gene identifier
**CAALFM_C114430CA**	**NADP-dependent oxidoreductase**	**EX2**	**NC_032089.1**	**3171401-3179800**	**Deletion**	**CNVs**
**CAALFM_C114440CA**	**GDI1: Rab GDP-dissociation inhibitor**					
**CAALFM_C114450CA**	**X-Pro aminopeptidase**					
**CAALFM_C114460WA**	**Metalloprotease**					
**CAALFM_C114470WA**	**Zinc-finger domain protein: mRNA splicing protein**					
**CAALFM_C210420WA**	**FGR6-4: Filamentous Growth Regulator**	**EX1; EX2; EX3**	**NC_032090.1**	**2152201-2154800**	**Duplication**	**CNVs**
**CAALFM_C202940WA**	**MET1: uroporphyrinogen-III C-methyltransferase**	**EX1; EX2**		**586701-588300**		
**CAALFM_C202950WA**	**Kynurenine–oxoglutarate transaminase**					
**CAALFM_C301250WA**	**PMC1 calcium-transporting ATPase**	**EX2**	**NC_032091.1**	**268201-269200**	**Deletion**	**CNVs**
**CAALFM_C307020WA**	**SSN6 transcription regulator**			**1611101-1611600**		
**CAALFM_C307030CA**	**Hypothetical protein**					
**CAALFM_C300030CA**	**Hypothetical protein**	**EX1**		**5100-7800**	**Duplication**	
**CAALFM_C604130CA**	**ALS4; GPI-anchored adhesin protein**	**EX1; EX2; EX3**	**NC_032094.1**	**899602-903604**	**Inversion**	**SVs**
				**899874-903095**	**Translocation**	
**CAALFM_C602720CA**	**Hypothetical protein**	**EX1**		**561312-564311**	**Deletion**	**CNVs**
**CAALFM_C603710WA**	**ALS9: GPI-anchored adhesin protein**	**EX2**		**799201-799500**		
		**EX3**		**800696-801497**		
**CAALFM_CR06660WA**	**SEO1: putative permease**	**EX1; EX2; EX3**	**NC_032096.1**	**1429763-1431288**	**Inversion**	**SVs**
**CAALFM_CR03620CA**	**Hypothetical protein**	**EX3**		**803187-803643**	**Deletion**	**CNVs**
**CAALFM_CR03630WA**	**IFF3: GPI-anchored protein**	**EX2; EX3**		**804801-805400**		

Furthermore, we identified other CNVs deletion encompassing genes of particular interest in chromosomes NC_032094.1 and NC_032096.1, which are involved in the process of adhesion to host surfaces. These finding implied that genomic variation in amoebae-exposed isolate could be affected on interaction with amoeba.

## 4 Discussion

Amoebae, as natural phagocytic micropredators, play a crucial role as fungal-predators, exerting selection pressure on the fungal organisms they ingest. These amoebae are not limited to the ingestion and intracellular killing of yeast cells or conidia. They also exhibit the ability to attack and ingest entire hyphae (Radosa et al., 2023). Thus, when considering that fungi and their amoeba predators may have interacted for eons, it appears obvious that predatory selection pressure could have had significant implications for fungal evolution. For example, the amoeba *P. aurantium* is commonly found on plant leaves. This amoeba species can prey on *C. parapsilosis*, an opportunistic fungal pathogen, and phagocytoses of the yeast cells induces an oxidative stress resulting in lysis of the yeast cells in acidified phagolysosomes. However, *C. parapsilosis* has evolved strategies to respond to reactive oxygen species (ROS) generated by predators. One notable response is the high expression of a *PRX1* gene by *C. parapsilosis* during interactions with amoebae. This gene encodes a thioredoxin-linked peroxidase, which is vital for cell redox homeostasis and the response to oxidative stress (Radosa et al., 2021). Moreover, other studies have revealed that *C. auris* can survive and proliferate within two amoebae species, *Vermamoeba* (*Hartmannella*) *vermiformis* and *A. castellanii*, suggesting that this environmental yeast has adapted to survive these fungal-predators (Hubert et al., 2021). Furthermore, a study investigating the interaction between *A. castellanii* and *C. neoformans* demonstrated that yeast cells survive following their interaction with amoebae and undergo morphogenic shifts from a yeast to pseudohyphae form. Additionally, these interactions displayed variable expression of virulence-associated traits, such as differences in capsule size (Chrisman et al., 2011), urease production, and melanization as well as affecting genetic variation (Fu et al., 2021). In the present study, we report the discovery that amoebae interactions modify the phenotype and genotype characteristics of environmental *C. albicans* strains.

In response to predation by *A. castellanii*, *C. albicans* strains isolated from diverse environmental sources commonly exhibited hyphal, pseudohyphal, and yeast formations. Different fungal morphologies are known to induce distinct killing mechanisms by the amoebae (Idnurm, 2023), suggesting that isolates generating hyphal forms may be resistant to predation. This finding corresponds to previous observations indicating that these morphological alterations serve as mechanisms for the fungi to evade and protect themselves from predation by fungal-predators. Specifically, *C. albicans* forms elongated hyphae and pseudohyphae when challenged with predation of *D. discoideum* as a soil-dwelling amoeba (Koller et al., 2016). These elongated structures play a crucial role in the ability of *C. albicans* to resist phagocytosis by amoebae and macrophages (Casadevall et al., 2019b). In the absence of interaction with amoebae, *C. albicans* typically presents as a yeast in serum-free PYG medium. Hyphal formation of *C. albicans* may be triggered by a secreted factor from staving amoeba cells (Koller et al., 2016), which is important for hyphal initiation in *C. albicans* (Liu et al., 1994). Interestingly, we observed that four *C. albicans* isolates derived from the “Flower (F)” reverted to yeast forms upon transfer to PYG medium without interacting with amoebae. Of our 20 exposed isolates, the morphology of sixteen isolates were maintained, exhibiting a stable hyphal and pseudohyphal morphology. For three isolates derived from “water hot spring (WH)”, whole genome sequencing identified insertion and deletion (Indels) in the *TUP1* gene. These genetic alterations resulted in the loss of an amino acid, glycine, in the TUP1 protein, which could alter gene function. A previous study reported that mutation of the *TUP1* gene induced filamentous formation in *C. albicans* (Braun and Johnson, 2000). This gene encodes a transcriptional repressor critical for negatively regulating filamentous growth in *C. albicans* (Kebaara et al., 2008). TUP1 interacts with the corepressor proteins SSN6 or TCC1, and these complexes function with DNA binding proteins to repress gene expression (Lee et al., 2015). On the other hand, the activation of TUP1 transcription repressor complexes leads to the repression of filament-specific gene expression (Kaneko et al., 2006; Kebaara et al., 2008). Further analysis is required to define the effects of the identified alterations in our exposed isolates. In addition, we detected CNV deletions between nucleotides 1,611,101 and 1,611,600 in the exonic regions of the *SSN6* and CAALFM_C301250WA genes in exposed isolates. This result is consistent with previous research demonstrating that alterations in *C. albicans SSN6* expression led to a loss of the capacity to optimize cellular morphology, and that the absence of the *SSN6* gene led to the formation of elongated filaments (Hernday et al., 2016). Hence, our findings suggest that the interaction of *C. albicans* with amoeba leads to the generation of filament forms, which are associated with genetic variations. However, the interaction with amoebae resulting in the mutation of the *FTH2* gene had no effect on their growth under iron depletion. Therefore, this phenomenon might not have a significant impact on iron metabolism. The high-affinity iron transport system of *C. albicans* includes key genes, including *FTR1*, *FTR2*, *FTH1*, *FTH2*, and *FET*. Cytoplasmic iron permease transporters, encoded by *FTR1* and *FTR2* genes, play an important role in acquiring iron from the environment. *FTH1* and *FTH2* serve as vacuolar membrane transporters, facilitating the transportation of iron from the vacuole to the cytoplasm during iron starvation. Previous research has reported that iron starvation conditions induced the highest expression of *FTR1*, *FTR2*, and *FTH1*. In contrast, *FTH2* exhibited constitutive expression in the presence or absence of iron levels (Mamouei et al., 2017). In addition, the deletion of the *ADE4* gene did not alter *Candida* growth in a medium without adenine, indicating that these isolates are not adenine auxotrophic strains. Although the mutation of the *ADE4* gene would be expected to adversely affect AMP biosynthesis, the amoebae-exposed isolates might grow on media without adenine by utilizing alternative pathways. In fact, *C. albicans* can generate AMP through three main processes: (1) the *de novo* purine biosynthesis pathway, (2) adenine salvage pathways, and (3) the S-adenosylmethionine (SAM) cycle. The *ADE4* gene serves as the first enzyme in the *de novo* purine biosynthesis pathway, converting phosphoribosyl pyrophosphate (PRPP) as a precursor to phosphoribosyl amine (PRA), which is the first intermediate product. Notably, glycine can substitute for PRA, leading to the generation of the next intermediate product for AMP production (Wangsanut et al., 2017). Moreover, adenosine from the SAM cycle is directly converted to AMP by ADO1 (Wierman et al., 2015). Taken together, the possible mechanism of AMP biosynthesis in *C. albicans* during amoeba interaction needs further investigation.

Because there are many different varieties of microorganisms in natural habitats, each has a unique set of traits and characteristics that aid in evading consumption by microbial predators. In fungi, for example, *Cryptococcus* produces a capsule (Araujo et al., 2012), whereas *Candida* has the ability to acquire iron (Gerwien et al., 2017) and produce biofilms (Pereira et al., 2021). These pathogens have interacted and adapted to survive interactions with amoebae, including by modifying responses to different stressors, including oxidative, cellular and thermal challenges. Thus, the exhibition of fungal virulence traits should also be considered in relation to the influence of amoeba predation on stressors and antifungal drug susceptibility (Casadevall et al., 2019b). Our findings suggest that most exposed isolates demonstrated enhanced survival under various stress conditions compared to the unexposed isolates. Notably, these phenotypic outcomes differ from those observed in a clinical isolate of *C. albicans* (Koller et al., 2016). Unfortunately, genomic analysis indicated no abnormalities in the stress response genes. It is probable that growth of *C. albicans* on a specific stressor is indirectly influenced by amoebae but directly induced by the stressor. The ability of the *C. albicans*-exposed isolates to resist thermal stress could, for example, involve the upregulation of heat shock protein (*HSP*) genes during high temperatures. Additionally, resistance to oxidative stress in the *C. albicans*-exposed isolate appears to be activated by both amoebae and oxidative stress. Our results are consistent with the study on the interaction between *C. albicans*, *C. parapsilosis, C. glabrata* with *P. aurantium* (Radosa et al., 2021). Furthermore, co-culture with amoeba resulted in some isolates demonstrating notable resistance against cell wall stressors and fluconazole, indicating increased stress tolerance compared to the unexposed isolates. This enhanced resistance can be attributed to the intricate interaction between *C. albicans* and amoeba, imposing selective pressure on the ingested yeast. Interestingly, we witnessed a marked increase in fluorescence intensity of calcofluor white (CFW) represented in septate hyphae, tips of hyphal cells, and mother cells in the amoebae-exposed isolate, indicating an increase in chitin content, compared to the unexposed fungal cells (**Supplementary Figure 5**). This implies that amoebae interactions might influence the chitin synthetic pathway in the yeast’s cell wall. Accordingly, these conditions might prompt yeast cells to enhance their cell walls by upregulating chitin synthesis, a process involvedly controlled by the *YCK2* gene (Caplan et al., 2020). These alterations in the cell wall are direct consequences of the diverse stressors encountered during the fungal predator-yeast interactions (Radosa et al., 2021).

Our findings also demonstrate that adaptation of *C. albicans* during co-culture with amoebae can result in significant alterations in their virulence factors, greatly affecting the fungus’ ability to form hyphae, invade, and kill A549 cells. The alterations in virulence are most striking for WH and S isolates, which displayed a substantial increase in their ability to generate hyphae and invade cells. Notably, interactions with amoebae resulted in the mutation of genes such as *SSN6*, which has been shown to control hyphal production in exposed isolates (Lee et al., 2015). In contrast to the amoebae-exposed isolates WH and S, amoebae-exposed isolate F displayed a high percentage of damaged A549 cells, but the isolate morphologically remained in yeast cell form.

This study emphasizes that *C. albicans* isolated from different ecological niches have the potential to adapt to various hostile conditions. This may partly explain why there are often reports of new emerging of *Candida* species that are opportunistic pathogens, promoting disease severity, enhancing antifungal resistance. This also supports the current hypotheses of “amoeboid predator-fungal animal virulence” and ‘‘environmental virulence school’’ (Casadevall et al., 2019b; Siscar-Lewin et al., 2022), especially as amoebae are similar in many behaviors to macrophages and adaptations to avoid killing by amoebae can also facilitate the pathogen subverting macrophage responses. The major limitation of this study is the co-culturing duration. Extending the co-culturing period or having intermittent periods of growth free of the predatory amoebae may exert a greater influence on the phenotypic and genotypic adaptations of *C. albicans* under elevated and prolonged stress conditions, potentially providing a better reflection of the reality of natural environments. We also only tested a limited set of *C. albicans* isolates, which may not be reflective of all environmental isolates.

## 5 Conclusion

**Figure 6:**
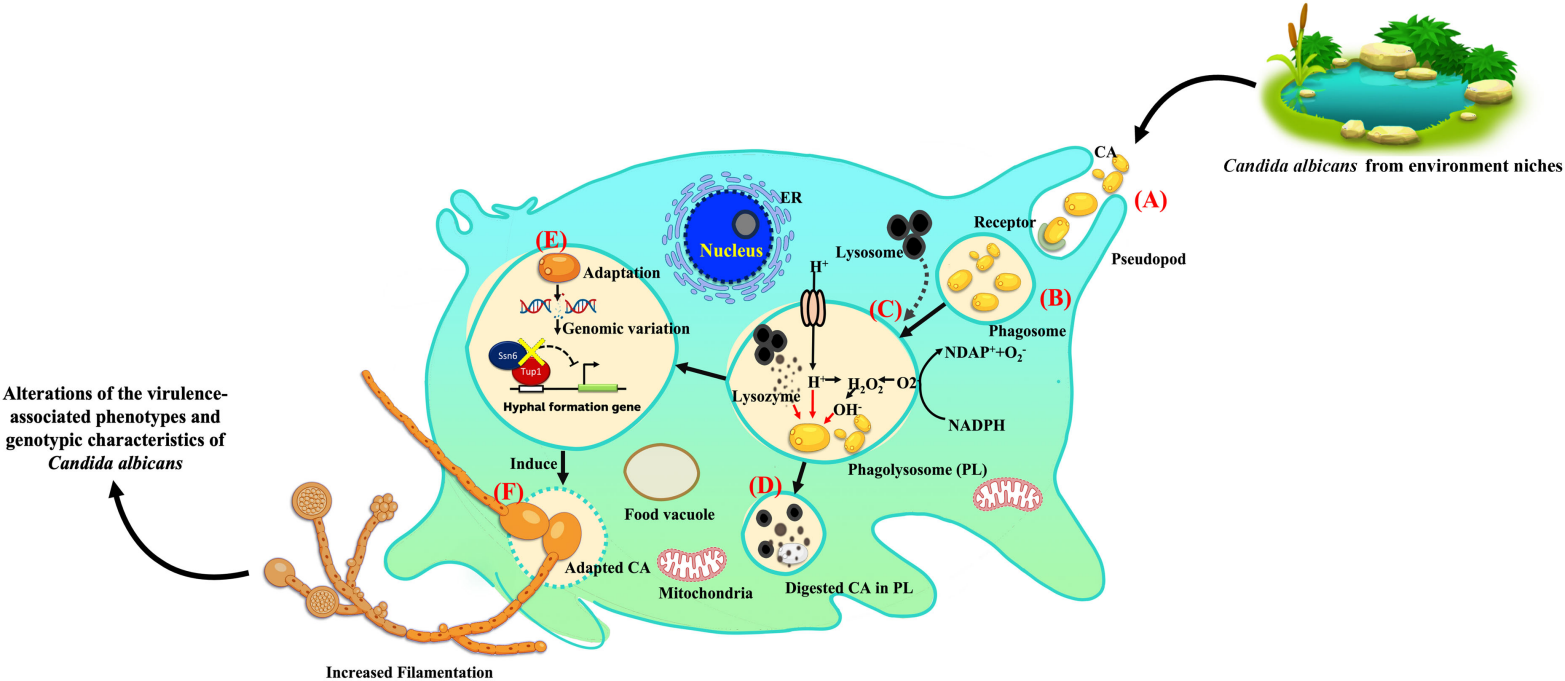
Proposed hypothetical model of amoeba-*Candida albicans* interaction and induced *C. albicans* adaptation. This proposed hypothetical model is a modification of previously described concepts (Novohradská et al., 2017; Rayamajhee et al., 2022). The stages of environmental *C. albicans* isolates engagement and entry into a predator and subsequent interactions are as follows: **(A)** uptake of extracellular *C. albicans*, primarily triggered by mannose-binding receptors after phagocytosis; **(B)** formation of an early phagosome; **(C)** phagosome fusion with lysosomes to form the phagolysosome (PL), which typically destroys engulfed yeast through low pH conditions and oxidative stress; **(D)** some yeast cells, unable to escape phagolysosome killing, are degraded; **(E)** yeast cells that can evade phagosome-lysosome fusion or resist antimicrobial factors in the phagosome escape lysosomal killing, indicating adaptation. **(F)** Genomic variations are also found during adaptations, including mutations in the *TUP1* and *SSN6* genes, leading to enhanced *C. albicans* filamentation. In summary, amoebae induce the alteration of the virulence-associated phenotypes and genotypic characteristics of *C. albicans* environmental isolates. Some icons included in this model were obtained from BioRender.

This study demonstrates that the complex interactions between *A. castellani* and environmental isolates of *C. albicans* can alter the biology of these yeast. Exposure to amoeba induces significant morphological changes in *C. albicans*, leading in particular to enhanced filamentation, a crucial virulence factor. Notably, observed mutations in the *TUP1* and *SSN6* genes might have contributed to the observed phenotypic alterations. The proposed hypothetical model of this study is illustrated to showcase the essential concept for future investigation (**Figure 6**). Overall, our findings suggest that the survival of *C. albicans* during amoeba predation is linked to the emergence of phenotypic and genetic alterations. These adaptations involve the development of mechanisms to enhance resistance to stressors and increase virulence associated traits, ultimately enhancing the probability for *C. albicans* to survive in diverse environmental niches and potentially within mammalian hosts.

## Data availability statement

The datasets presented in this study can be found in online repositories. The names of the repository/repositories and accession number(s) can be found below: The datasets of *C. albicans* described in this paper have been deposited in the National Center for Biotechnology Information GenBank database under the BioProject number PRJNA1065157.

## Ethics statement

Ethical approval was not required for the studies on humans in accordance with the local legislation and institutional requirements because only commercially available established cell lines were used. The manuscript presents research on animals that do not require ethical approval for their study.

## Author contributions

AA: Conceptualization, Data curation, Formal analysis, Funding acquisition, Investigation, Methodology, Visualization, Writing – original draft. KP: Conceptualization, Data curation, Formal analysis, Investigation, Methodology, Validation, Writing – original draft. PT: Conceptualization, Data curation, Investigation, Validation, Writing – review & editing. JN: Data curation, Investigation, Validation, Formal analysis, Supervision, Writing – review & editing. SY: Data curation, Investigation, Supervision, Validation, Writing – review & editing, Conceptualization, Funding acquisition, Resources, Visualization.
