# Supplementary figures and images for: Adaptation to an amoeba host drives selection of virulence-associated traits and genetic variation in saprotrophic Candida albicans
Source: Front Cell Infect Microbiol. 2024 Mar 13;14:1367656. doi: 10.3389/fcimb.2024.1367656 (PMC10976851; doi:10.3389/fcimb.2024.1367656)

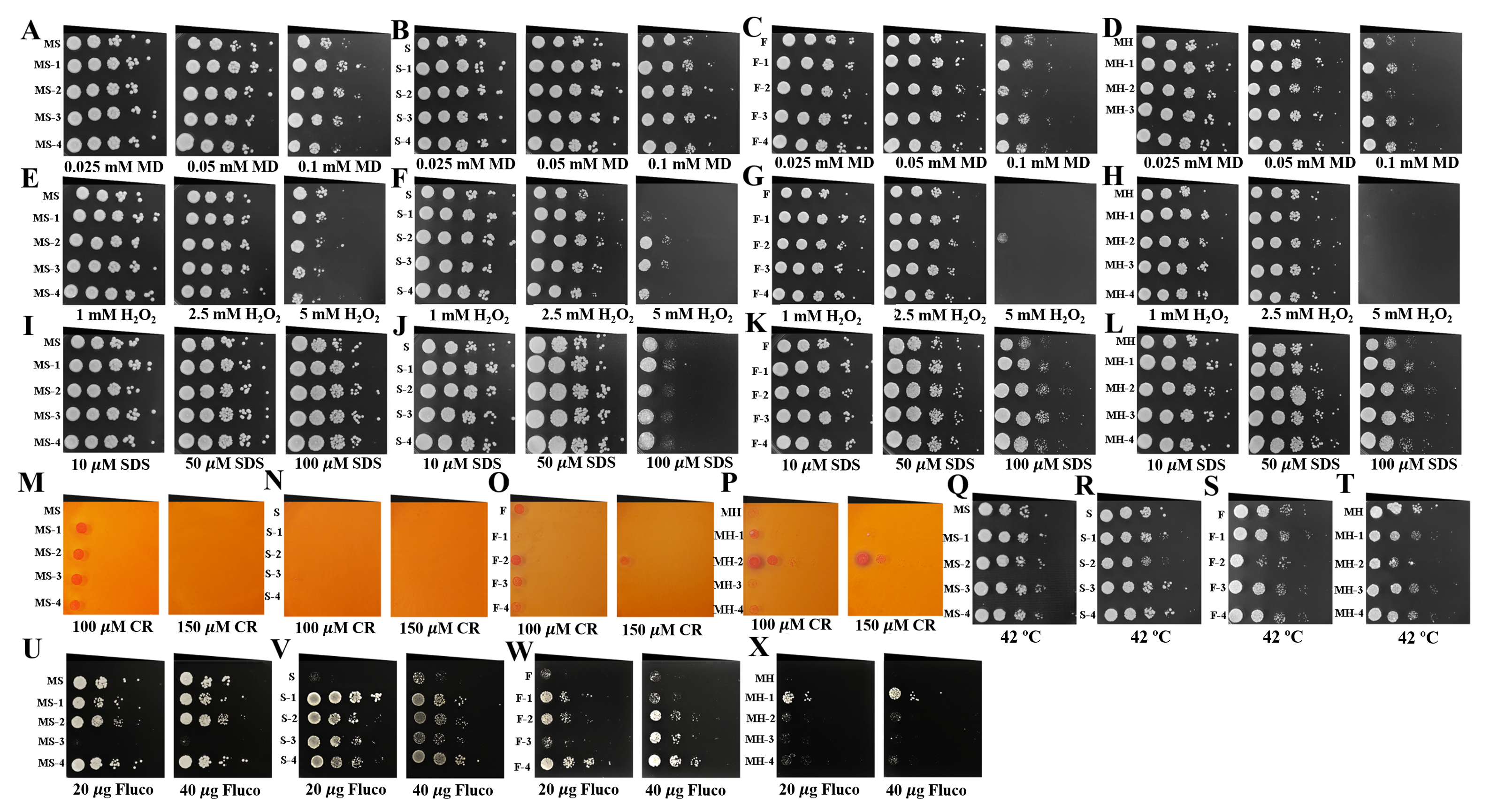

Supplement: Supplementary file 1 [file DataSheet_1.zip › Supplement_figure_1.tif]

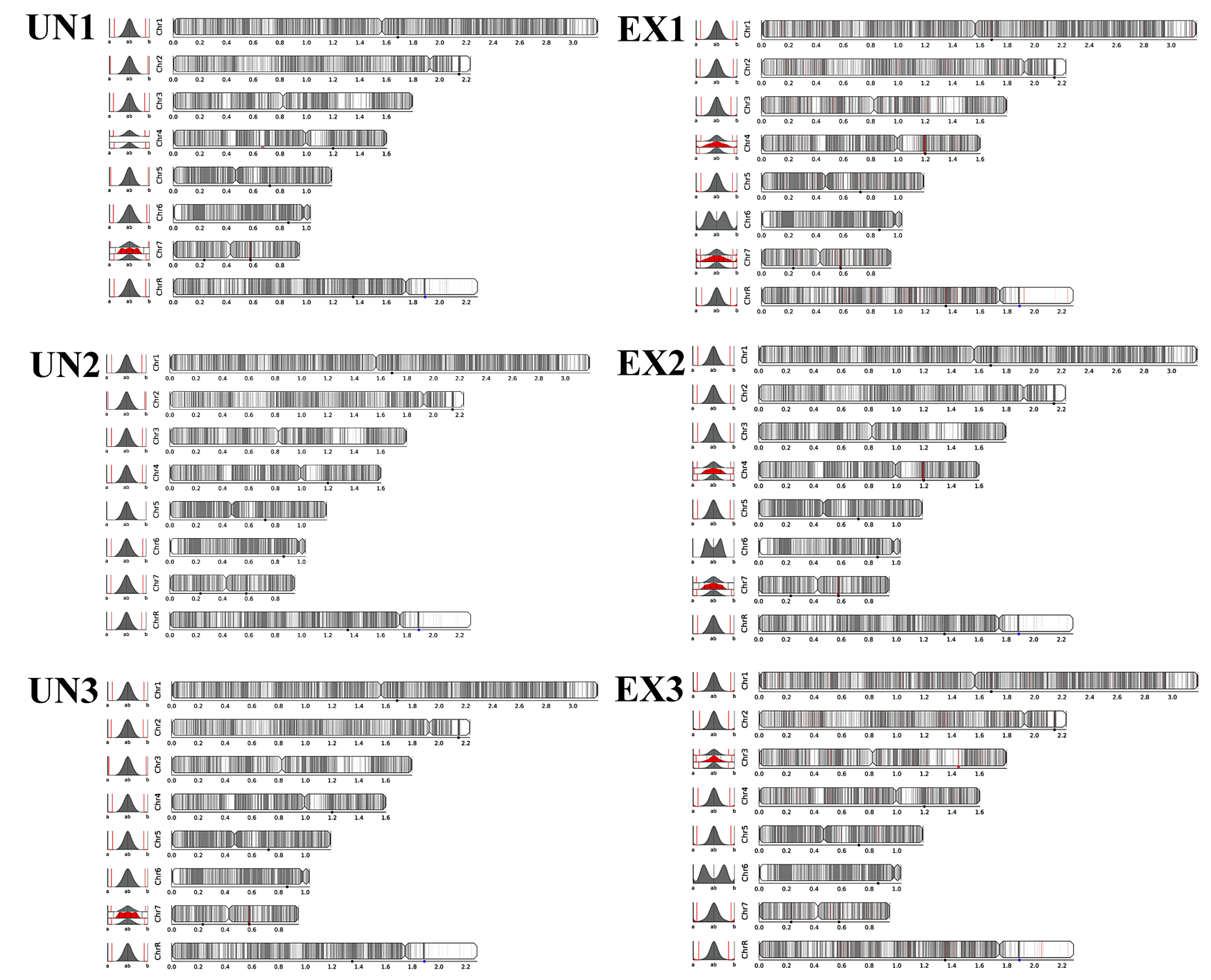

Supplement: Supplementary file 1 [file DataSheet_1.zip › Supplement_figure_2.tif]

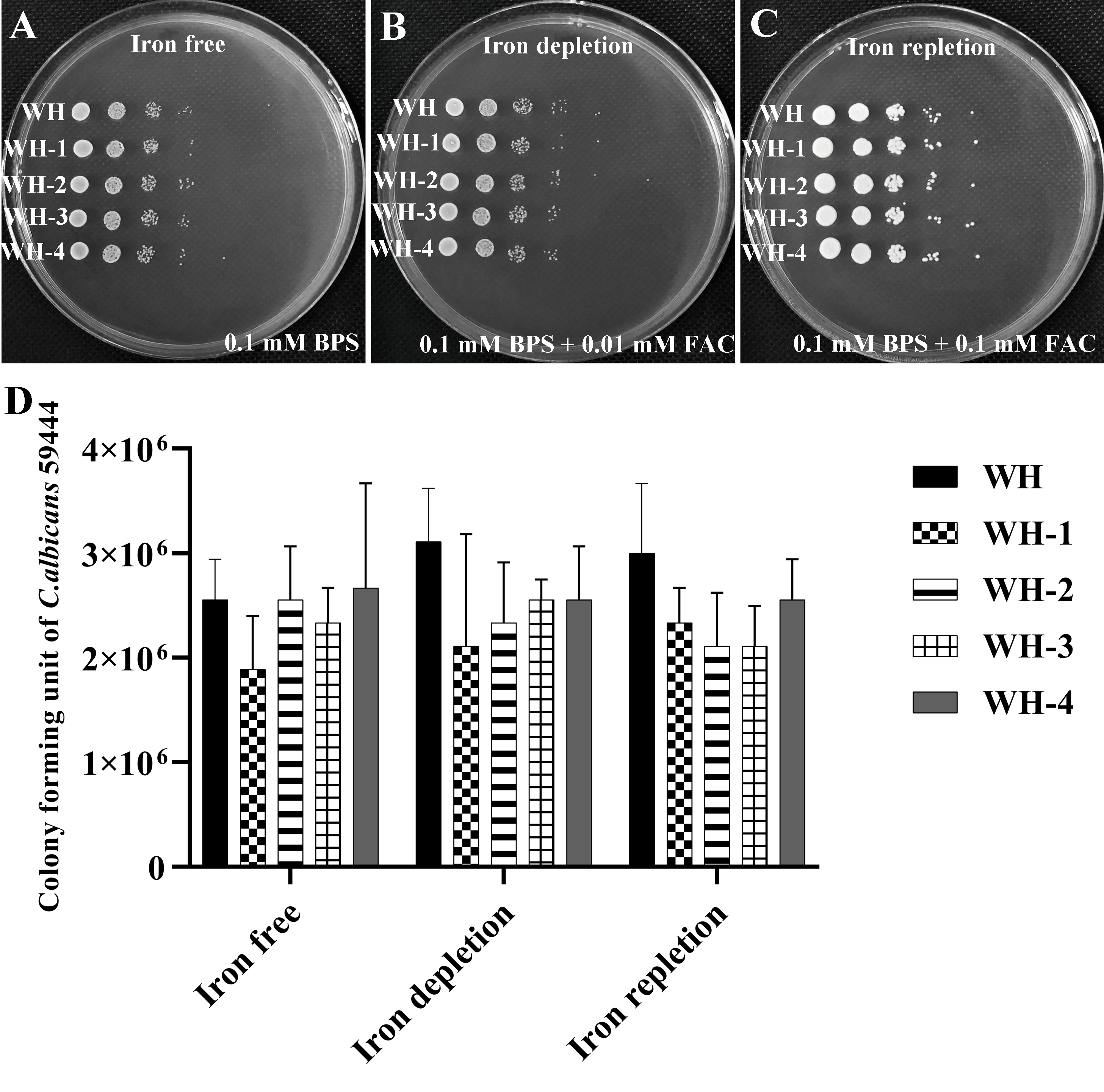

Supplement: Supplementary file 1 [file DataSheet_1.zip › Supplement_figure_3.tif]

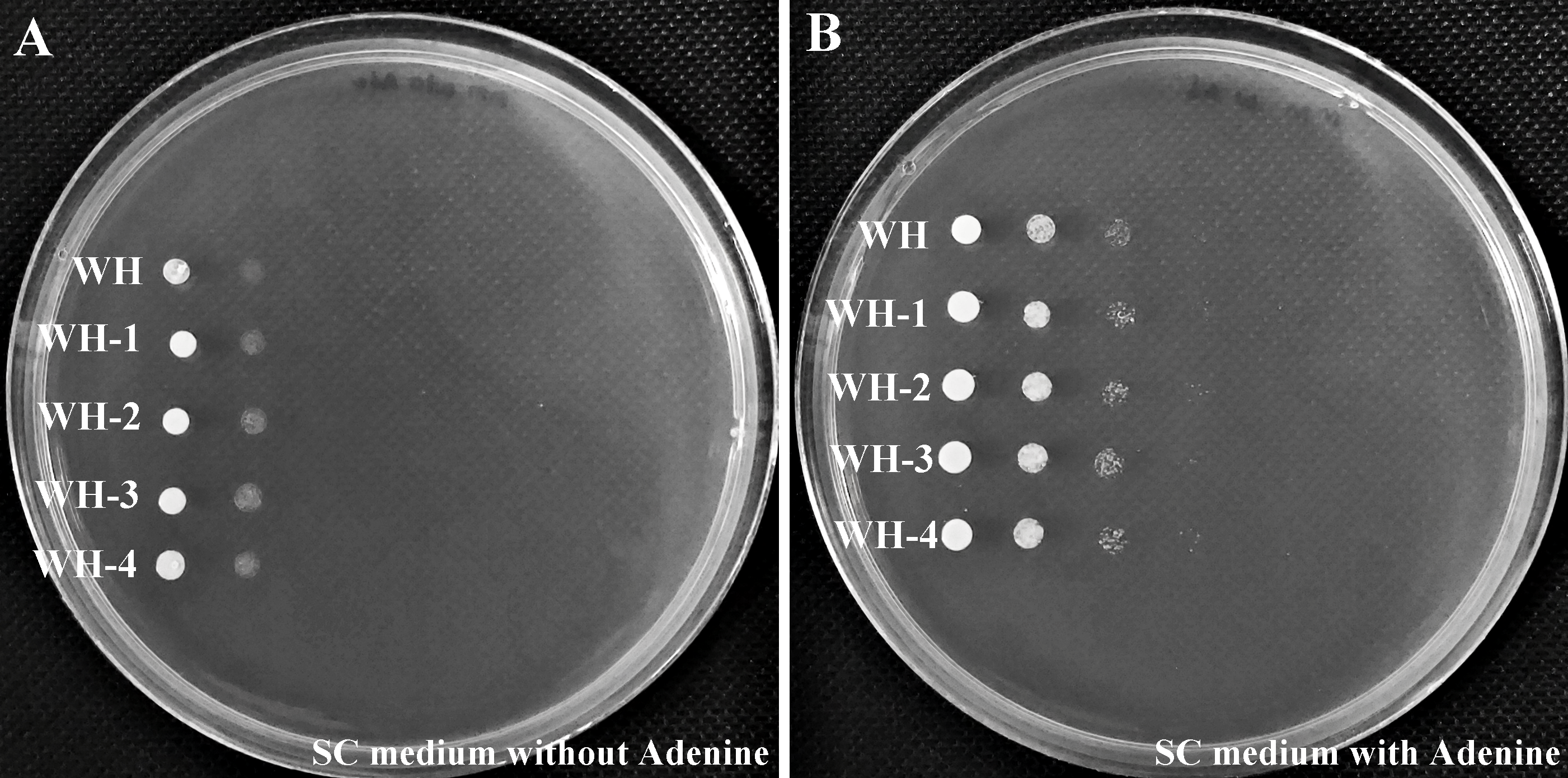

Supplement: Supplementary file 1 [file DataSheet_1.zip › Supplement_figure_4.tif]

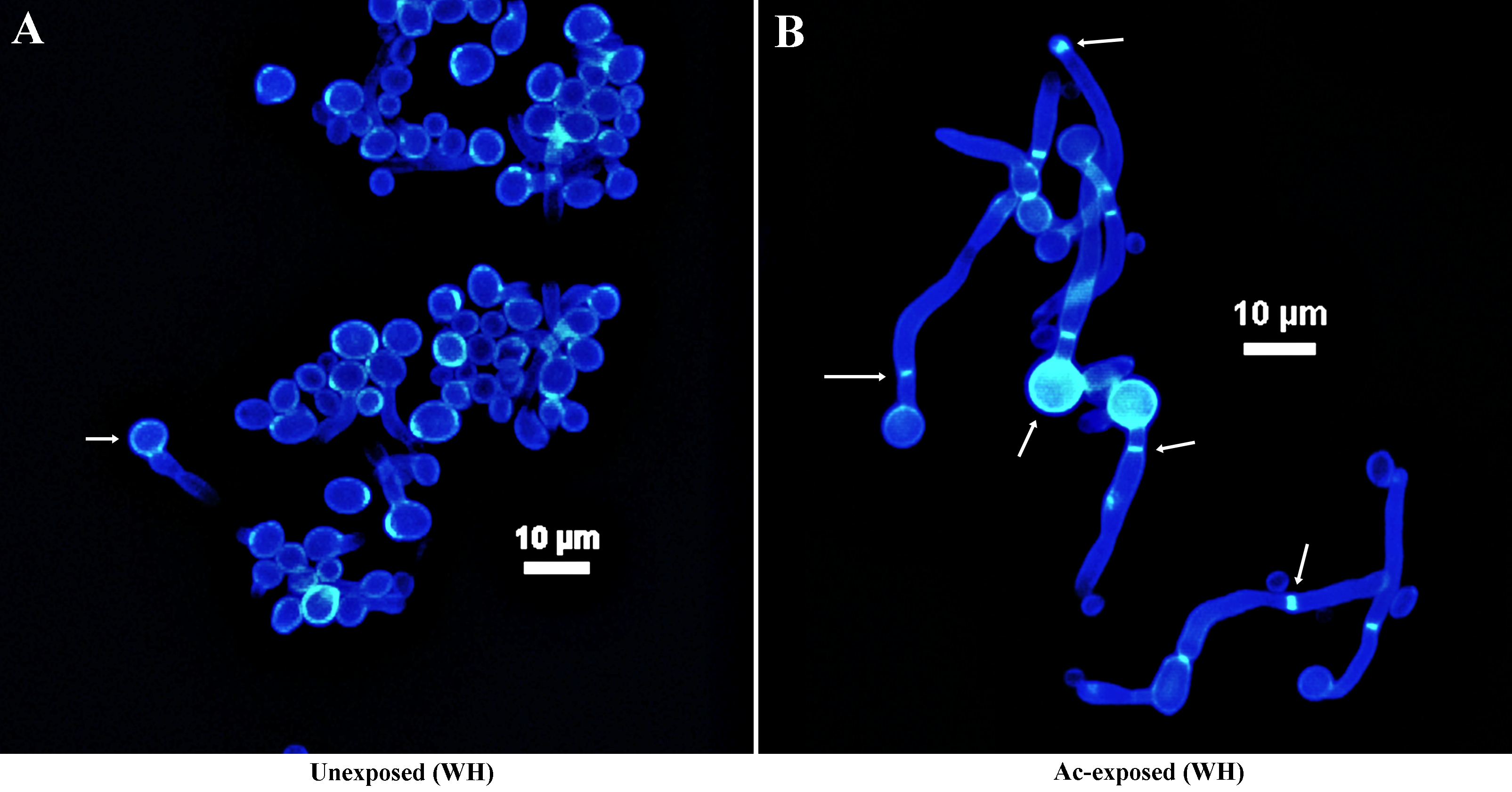

Supplement: Supplementary file 1 [file DataSheet_1.zip › Supplement_figure_5.tif]
