# Supplementary material for: Adaptation to an amoeba host drives selection of virulence-associated traits and genetic variation in saprotrophic Candida albicans
Source: Front Cell Infect Microbiol. 2024 Mar 13;14:1367656. doi: 10.3389/fcimb.2024.1367656 (PMC10976851; doi:10.3389/fcimb.2024.1367656)
Supplement: Supplementary file 2 [file Table_1.docx]

Supplementary Material

# Supplementary Figures and Tables

## Supplementary Figures

**Supplementary Figure 1. Phenotypic stress responses of *C. albicans* under various stress conditions (the fungal isolated from Moss (MS), Sediment (S), Mushroom (MH), and Flower (F))**. To test growth of amoebae exposed isolates, comparing unexposed isolate under various stressors. These figures present an investigation into the growth of parental and adapted isolates under stress conditions induced by menadione **(A-D)** and hydrogen peroxide **(E-H)** as oxidative stress, sodium dodecyl sulfate (SDS) **(I-L)** and Congo red **(M-P)** as cell wall stress, incubation at 42 °C as thermal stress **(Q-T)**, and an investigation of amoeba influence on adapted isolates to resist fluconazole **(U-X)**.

**Supplementary Figure 2. Whole genome sequence data plotted of *C. albicans* using YMAP.** To analyze a genetic variation in *C. albicans* (WH) genome after amoeba interaction by YMAP. YMAP plots illustrate the genome-wide scale changes occurring in AC-exposed isolates (EX1-3) compared to AC-unexposed isolates in *C. albicans* strain WH. The YMAP plots consist of gray lines indicating an equal representation of both alleles (A and B) of each gene across the chromosomes, with the red line highlighting different SNPs/LOH.

**Supplementary Figure 3. *FTH2* gene mutation does not have any effect on iron starvation.** To examine the effect of the *FTH2* gene mutation in exposed isolates on growth under iron free, iron depletion, and iron repletion conditions. The growth of unexposed strains and exposed isolates under iron free **(A)** and iron depletion **(B)** and iron repletion **(C)**. Cells were 10-fold serially diluted (10^6^ to 10^1^) and spotted onto PYG medium was supplemented with 100 μM bathophenanthroline (BPS) to chelate iron ions (Fe^2+^ or Fe^3+^) in the media, creating an iron free condition. For the iron depletion, PYG medium with 100 μM BPS was supplemented with 0.01 mM ferric ammonium citrate (FAC), and the iron repletion was added with 0.1 mM FAC. The plates were incubated at 28 ℃ for 24 hours. Colony forming units (CFU)/mL were then determined at a dilution of 10^3^ cells/mL. Colonies were photographed after 24 hours of inoculation. Amoeba-passaged isolates were labeled with the numbers as WH1-4, whereas WH was presented as unexposed isolate. The statistic was calculated by two-way ANOVA in conjunction with Tukey's multiple comparison test.

**Supplementary Figure 4. *ADE4* gene mutation isolate is not an adenine auxotroph.** To examine the effect of the *ADE4* gene mutation in exposed isolates on growth in YNB, with (right) or without adenine (left). The growth of unexposed strains and exposed isolates under SC medium without adenine **(A)** and with adenine **(B)**. Cells were 10-fold serially diluted (10^6^ to 10^1^) and spotted onto SC+Ade or SC-Ade, incubated at 28 ℃ for 24 hours to observe growth.

**Supplementary Figure 5. Amoebae interaction represented an increase in chitin content.** To assess the effect of amoebae interaction on the chitin content of exposed isolate compared to unexposed isolate of WH. Yeast cells were grown in the PYG broth for 24 hours, exhibiting both yeast and hyphal forms. Then, cells were strained with calcofluor white (CFW) at a concentration of 0.1 mg/mL to assess chitin content in yeast cells. The fluorescence intensity of calcofluor white

was observed between unexposed **(A)** and exposed **(B)**. White arrow presented the location of chitin deposition.

# 1.2 Supplementary Tables

**Supplementary Table 1. Analysis of SNPs in the *C. albicans* genome after amoeba interaction.** This table presents the distribution of the SNP type and the SNP on the location of sample genomes, comparing among AC-exposed isolates (EX1-3) and AC-unexposed isolates (UN1-3).

**Supplementary Table 2. Analysis of Indels in the *C. albicans* genome after amoeba interaction.** This table presents the distribution of Indels on the location of sample genomes, comparing among AC-exposed isolates (EX1-3) and AC-unexposed isolates (UN1-3).

**Supplementary Table 3. Analysis of SVs in the *C. albicans* genome after amoeba interaction.** This table presents the distribution of SVs on the location of sample genomes, comparing among AC-exposed isolates (EX1-3) and AC-unexposed isolates (UN1-3).

**Supplementary Table 4. Analysis of SVs length in the *C. albicans* genome after amoeba interaction.** This table presents the distribution of SVs length on the sample genomes, comparing among AC-exposed isolates (EX1-3) and AC-unexposed isolates (UN1-3).

**Supplementary Table 5. Analysis of CNVs in the *C. albicans* genome after amoeba interaction.** This table presents the types of CNVs on the sample genomes, comparing among AC-exposed isolates (EX1-3) and AC-unexposed isolates (UN1-3).

**Supplementary Table 6. Analysis of impacts SNPs and Indels in the *C. albicans* genome after amoeba interaction.** This table presents the impacts of SNPs and Indels on each sample genome, comparing among AC-exposed isolates (EX1-3) and AC-unexposed isolates (UN1-3).

**Supplementary Table 7. Analysis of impacts SVs in the *C. albicans* genome after amoeba interaction.** This table presents the impacts of SVs on each sample genome, comparing among AC-exposed isolates (EX1-3) and AC-unexposed isolates (UN1-3).

**Supplementary Table 8. Analysis of impacts CNVs in the *C. albicans* genome after amoeba interaction.** This table presents the impacts of CNVs on each sample genome, comparing among AC-exposed isolates (EX1-3) and AC-unexposed isolates (UN1-3).
